# Mapping the non-coding RNA landscape in ataxia telangiectasia: a scoping review of ATM dependent miRNA and lncRNA dysregulation

**DOI:** 10.1007/s11033-025-11094-x

**Published:** 2025-10-09

**Authors:** Muhammad Junaid Iqbal, Usman Ameen, Hamza Tanveer, Laraib Israr, Anastasia Ricci, Gianluca Morganti, Muhammad Jawad Khan, Michele Menotta

**Affiliations:** 1 Department of Biomolecular Sciences, University of Urbino “Carlo Bo”, Via Saffi 2, Urbino (PU), 61029 Italy; 2 Khyber Medical University, Peshawar, 25100 Pakistan; 3 Riphah Institute of Pharmaceutical Sciences, Riphah International University, Islamabad, 44000 Pakistan; 4 Department of Biological Sciences, International Islamic University Islamabad, Islamabad, 44000 Pakistan; 5 Department of Biosciences, COMSATS University Islamabad, Park Road, Tarlai Kalan, Islamabad, 45550 Pakistan

**Keywords:** Ataxia telangiectasia, Ataxia telangiectasia mutated proteins, MicroRNAs, Long non-coding RNA, DNA damage response, ATM

## Abstract

Ataxia-Telangiectasia (A-T) is caused by biallelic Ataxia-Telangiectasia Mutated (ATM) protein mutations and is hallmarked by neurodegeneration, immunodeficiency and cancer susceptibility. Emerging work suggests that non-coding RNAs are integral to the ATM driven DNA damage response, yet the scope of non-coding RNA perturbation in A-T has not systematically mapped. This study is done to chart and synthesize all primary evidence on ncRNA dysregulation in A-T and to identify mechanistic gaps and research priorities. The evidence base comprises a total of five studies focused exclusively on microRNAs and long non-coding RNAs. Baseline sequencing of 20 patients revealed consistent downregulation of miR-195-5p, miR-30a-5p and miR-342-3p. Low dose γ-irradiation of ATM-null lymphoblastoid cell lines produced an exaggerated bidirectional shift (8 microRNAs upregulated, 6 downregulated) versus wild-type, whereas primary T-cells showed largely ATM independent rises of miR-34a-5p and miR-182-5p. A lymphoblastoid cell lines study distinguished 22 recessive or dominant radiation-responsive microRNAs. Long non-coding RNAs profiling identified 149 transcripts that require functional ATM for induction and three uniquely upregulated in A-T cells. No circular RNA study in A-T is observed till date. The ncRNA research in A-T is nascent with five studies present a miRNA dysregulation and an ATM-dependent lncRNA surge that is lost in A-T, but leave circRNAs, neuronal tissues and functional validation unexplored. Intermediate dose stress models, total and small RNA-seq under DNA damage and oxidative stress, and CRISPR rescue/knockout screens in patient derived organoids are immediate priorities for exploring the role of ncRNAs as therapeutic targets in A-T.

**Supplementary Information:**

The online version contains supplementary material available at 10.1007/s11033-025-11094-x.

## Introduction

Ataxia Telangiectasia (A-T) is an uncommon genetic disease that leads to several other diseases such as oculocutaneous telangiectasia, sensitivity to radiation, immunodeficiency, progressive cerebellar neurodegeneration and high chances of getting cancers [1–5]. It arises due to biallelic mutations in the *ATM* gene. This gene translates into ATM kinase protein needed for DNA damage response. ATM gets activated in response to DNA double stranded breaks [1, 6]. Upon activation, ATM phosphorylates various downstream targets including p53, H2AX and MEF2D thereby coordinating cell-cycle checkpoints, DNA repair, apoptosis and redox homeostasis [7–9]. Loss of ATM function leads to chromosomal instability, radiosensitivity and an elevated cancer risk [10, 11]. Despite decades of research, no curative therapy exists, and current management is largely supportive. In recent trials, very low dose corticosteroids have demonstrated sustained clinical benefits, and the largest trial Ataxia Telangiectasia Treatment with EryDex System (ATTeST) did not meet its primary efficacy endpoint but the subgroup analysis on patients aged 6–9 showed a positive response. The study is still ongoing on this age group top map the response of intra-erythrocyte dexamethasone sodium phosphate [12–14].

Although A-T phenotypes are explained by protein centered models, recent studies have also shown the role of non-coding RNAs (ncRNAs) in A-T pathogenesis [15–17]. Multiple classes of ncRNAs affects ATM dysregulation due to the DNA damage response (DDR) [18–21] but only one study has reported the dysregulation of ncRNAs at basal level in the context of A-T [16]. The ncRNAs are functional RNA molecules that do not encode proteins. They are broadly categorized into smaller species such as microRNAs (miRNAs), circular RNAs (circRNAs), long ncRNAs (lncRNAs) [22–24]. miRNAs (~ 22 nt) generally repress gene expression post-transcriptionally by binding the 3′ untranslated region (UTR) of target mRNAs [25–27]. lncRNAs (>200 nt) act at chromatin, transcriptional and post-transcriptional levels [28, 29].

Both miRNAs and lncRNAs participate in DNA repair, genome stability and cellular stress responses, and several are induced by genotoxic stress in an ATM-dependent manner [17, 19, 30]. CircRNAs can sequester miRNAs or serve as protein scaffolds. Notably, a circRNA derived from ATM (circATM) was recently shown to regulate oxidative stress, although circRNA profiles in A-T have yet to be explored [31]. Although ATM has been extensively studied in DNA damage repair and cancer biology, little is known about the specific contribution of non-coding RNAs to A-T pathogenesis. Only five studies to date have profiled miRNAs or lncRNAs in patient-derived cells, and none have investigated circRNAs, snoRNAs, piRNAs, or neuronal and muscular tissues most affected in A-T. This lack of systematic synthesis has left unclear which ncRNAs are reproducibly dysregulated in A-T and how they might serve as biomarkers or therapeutic entry points. The objective of this review is to gather information of every discovered non-coding RNA in Ataxia Telangiectasia whether in samples or ATM deficient cell lines of A-T patients to create an up-to-date map of evidence that researchers can see in future of their dysregulation which can help in deciding that how future work should be focused on them in context of A-T.

## Methods

### Protocol and registration

A protocol with proper objectives, eligibility criteria, and search strategy was developed in March 2025. It was prospectively registered on the Open Science Framework (OSF) on 20 June 2025 (10.17605/OSF.IO/9W2KZ). The reporting follows the PRISMA Extension for Scoping Reviews (PRISMA-ScR) [32, 33]. The full checklist is provided in Supplementary File S1.

### Eligibility criteria

Eligibility criteria were framed with the Population, Concept and Context (PCC) (Table 1).

**Table 1:** Selection criteria of studies

Domain	Inclusion criteria	Exclusion criteria
Population	Human patients with a clinical and/or molecular diagnosis of Ataxia Telangiectasia (A-T). Primary or immortalized cell lines derived from A-T patients.	
Concept	Experimental measurement of non-coding RNAs (microRNAs, lncRNAs, circRNAs).	
Context	Studies compare A-T samples with healthy human controls, wild type (ATM-proficient) cell lines, or ATM null A-T cells.	
General		Reviews, editorials and conference abstracts (excluded from primary synthesis) • Full text not available • Publications in languages other than English. Purely mechanistic studies performed solely in relation to ATM in some other diseases other than A-T (retained for background discussion).

### Information sources and search strategy

To investigate current knowledge on role of ncRNAs in A-T, we conducted a scoping literature review following PRISMA guidelines for scoping reviews [32, 33]. We searched PubMed, Scopus, Embase, and Web of Science using relevant keywords of *“ataxia telangiectasia”* combined with *“microRNA” OR “lncRNA” OR “circRNA” OR “non-codingRNA”*. The search was limited to articles published in English, spanning from database inception to April 2025. Full search strings are provided in Supplementary File 2.

### Selection of sources of evidence

**Fig. 1:**
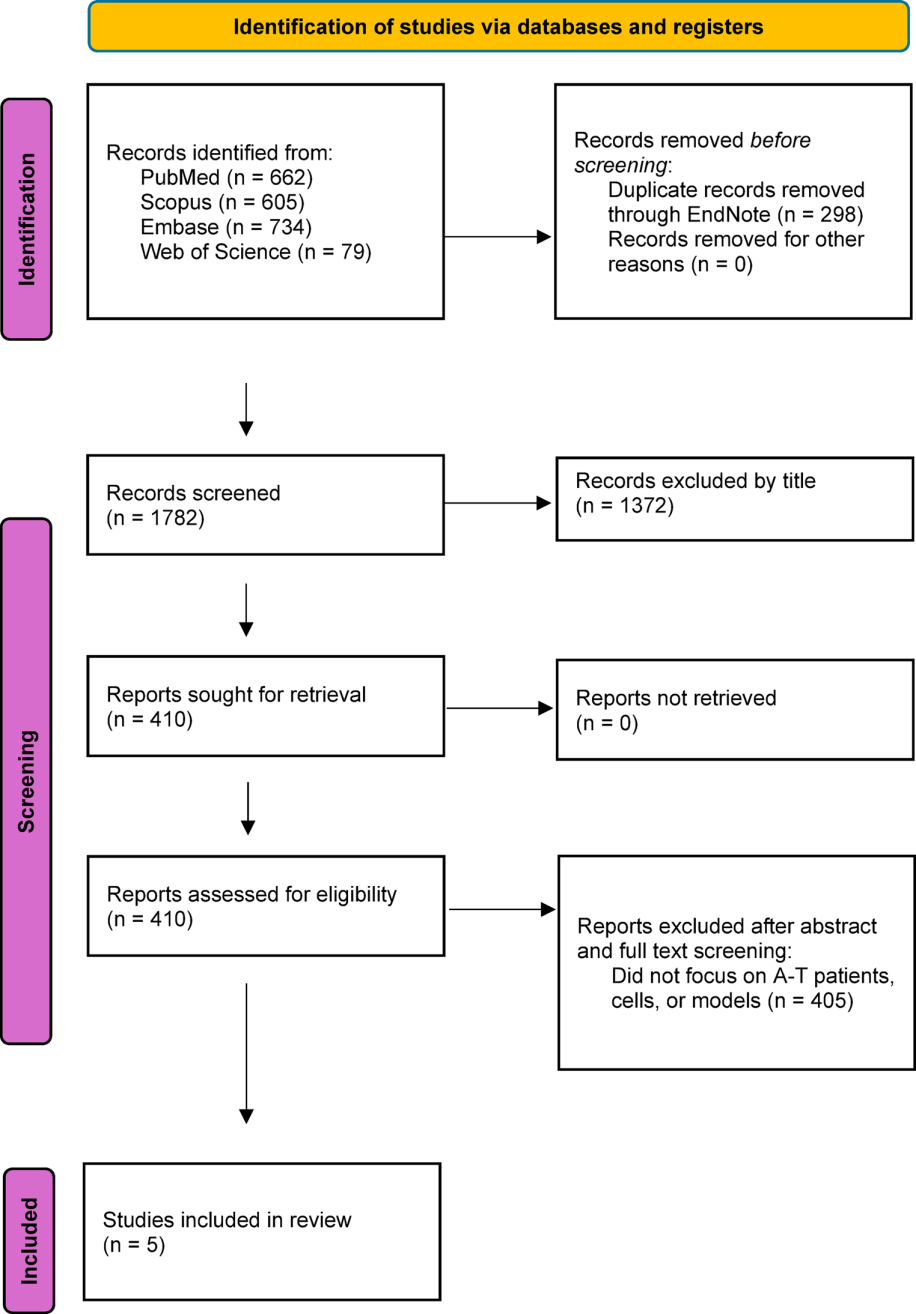
PRISMA flow diagram illustrating the selection process of studies included in the review of ncRNAs in A-T (Made in MS Word)

Articles were imported into EndNote and then duplicates were removed. Two reviewers screened the studies primarily on EndNote through title and successfully passed studies imported to Rayyan for secondary screening through abstract and then full text. To address any conflicts in both stages, a third reviewer was consulted. Articles that successfully passed the full text screening were selected for data extraction. The selection process is summarized in a PRISMA flow diagram in Fig. 1.

### Data charting and extraction

Data was extracted from five eligible studies by two reviewers independently. Discrepancies were resolved by consensus. A third reviewer was consulted to cross check the extracted data against the original publications. For each eligible study, different variables were extracted like the bibliographic details, the sample model (patient blood, tissue or specific A-T or wild-type cell lines together with any radiation dose or other experimental treatment), the type and name of ncRNA that is being investigated (miRNA, lncRNA or circRNA), and the direction of differential expression relative to healthy controls.

### Synthesis of results

Data were synthesized in a manner that different types of ncRNAs were discussed independently. Findings of ncRNA species, pattern of dysregulation and experimental model are presented in Tables 2 and 3. The synthesis in results was focused on miRNA expression pattern in A-T versus healthy controls, baseline miRNA dysregulation in patient tissues, radiation responsive miRNA expression pattern in cellular models, and ATM-dependent lncRNA responses. Narrative synthesis in discussion was focused on the comparative analysis of some ncRNAs found in A-T and their cross-disease context.

## Results

### Study selection and characteristics

A total of 2080 articles were identified through database searches in which 298 were excluded as duplicates and 1372 were removed after primary screening through titles on EndNote. Later the approved studies were imported into Rayyan where 405 studies were excluded following abstract and full-text screening because of ineligibility (focus not on ataxia telangiectasia), leaving five articles for analysis. The flow diagram is based on the 2020 version [33] of the PRISMA flow diagram (Fig. 1).

### MiRNA expression pattern in A-T versus controls

There are only four studies which have evaluated the miRNAs response in A-T till date. Cirillo et al. analyzed peripheral blood mononuclear cells (PBMCs), fibroblasts through NGS and total white blood cells (WBCs) for qRT-PCR validation under basal conditions, providing a reference set of baseline miRNA expression [16]. Bryant et al. profiled ATM-null and wild-type lymphoblastoid cell lines (LCLs) both at baseline and after a modest 0.05 Gy and 0.5 Gy dose of γ-irradiation, allowing comparison of baseline ± low-dose ionizing radiation (IR) states [15]. In contrast, Kabacik et al. focused on primary T-lymphocytes from an A-T patient versus two healthy donors characterizing a radiation inducible miRNA response [30]. Finally, Smirnov et al. examined EBV-transformed LCLs from controls, heterozygous carriers and A-T patients at 0 h, 4 h and 24 h after 5 Gy, defining miRNAs with either *recessive* or *dominant* IR-response phenotypes [34]. Together these studies span baseline dysregulation and stimulus-dependent miRNA dynamics across multiple patient-derived models. The details are also available in Table 2.

#### Baseline MiRNA dysregulation in patient tissues

Cirillo et al. performed short ncRNA seq analysis in 20 A-T patients by peripheral blood mononuclear cells (PBMCs) and three additional skin derived fibroblasts. They compared them with age matched healthy controls. RNA sequencing revealed 42 differentially expressed miRNAs in PBMCs (16 upregulated and 26 downregulated), and 26 differentially expressed miRNAs in fibroblasts (13 upregulated and 13 downregulated). This review only enlisted three miRNAs (Table 2) which stood out for their consistent downregulation and were also validated through WBCs. The miR-195-5p was showing significant downregulation in both PBMCs and fibroblasts but its downregulation was not significant in qRT-PCR analysis in white blood cells. The miR-30a-5p showed downregulation in PBMC RNA-seq and was further validated in WBCs samples by qRT-PCR giving similar outcome. miR-342-3p exhibited a non-significant downregulation in PBMCs but was confirmed as significantly downregulated in WBC qRT-PCR samples [16].

#### Radiation-responsive MiRNA expression pattern in cellular models

Bryant et al. examined 752 microRNAs in two ATM null lymphoblastoid cell lines (AT2Bi, AT3Bi) and an ATM proficient healthy control (2139) with dose of 0.05 Gy and 0.5 Gy irradiation. Eight miRNAs (miR-135a-5p, miR-152-3p, miR-223-3p, miR-328-3p, miR-424-5p, miR-618, miR-92a-1-5p, miR-99a-5p) were upregulated in both A-T lines whereas six (miR-138-5p, miR-141-3p, miR-181d-5p, miR-335-3p, miR-501, miR-497-5p) were downregulated relative to control as shown in Table 2 [15]. In another study, Kabacik et al. evaluated the ncRNA response in stimulated T-lymphocytes of human in a time and dose dependent manner. He performed experiments on two controls and 1 A-T cell line and irradiated them. They found out that out of 19 miRNAs, only miR-34a-5p and miR-182-5p rose with time. The strange finding was that there was almost no difference of expression between A-T and control samples, therefore, these miRNAs cannot be taken as a signature for diagnostic purposes for ATM deficiency [30].

Smirnov et al. profiled 157 miRNAs in EBV transformed lymphoblastoid cell lines (LCLs) from eight controls (ATM +/+), eight heterozygous carriers (ATM +/–) and eight A-T patients (ATM –/–). Cells were irradiated and sampled at 0 h, 4 h and 24 h. If miRNAs were significantly dysregulated (*p* < 0.001) in A-T but not in carriers and controls, they termed it as recessive pattern. While if the dysregulation is significant (*p* < 0.001) in A-T and carriers as compared to control, then it was a dominant pattern. There were 22 miRNAs reported to show recessive and dominant patterns. All the recessive (15 miRNAs) and dominant (7 miRNAs) patterns are enlisted in Table 2 [34]. It is important to note that many of these observations were derived from EBV-transformed lymphoblastoid cell lines (LCLs). Because LCLs are immortalized and proliferative by design, their baseline and stress response miRNA profiles may not fully represent physiological states. Differences between LCLs and primary samples likely reflect both ATM deficiency and model specific features.

**Table 2:** List of dysregulated MiRNAs in ataxia telangiectasia

Non-coding RNA	Expression pattern	Subjects/model	References
miR-195-5p	Downregulated	PBMC, and fibroblast	[16]
miR-30a-5p	Downregulated	PBMC, WBC	[16]
miR-342-3p	Downregulated	WBC	[16]
miR-205	Recessive and downregulated	EBV-LCLs, 5 Gy IR	[34]
miR-195	Recessive and upregulated	EBV-LCLs, 5 Gy IR	[34]
miR-200b	Recessive and upregulated	EBV-LCLs, 5 Gy IR	[34]
miR-199b	Recessive and upregulated	EBV-LCLs, 5 Gy IR	[34]
miR-181c	Recessive and upregulated	EBV-LCLs, 5 Gy IR	[34]
miR-190	Recessive and upregulated	EBV-LCLs, 5 Gy IR	[34]
miR-181a-2	Recessive and upregulated	EBV-LCLs, 5 Gy IR	[34]
miR-199a-2	Recessive and upregulated	EBV-LCLs, 5 Gy IR	[34]
miR-320	Recessive and upregulated	EBV-LCLs, 5 Gy IR	[34]
miR-339	Recessive and upregulated	EBV-LCLs, 5 Gy IR	[34]
miR-182	Recessive and upregulated	EBV-LCLs, 5 Gy IR	[34]
miR-16	Recessive and upregulated	EBV-LCLs, 5 Gy IR	[34]
miR-20a	Recessive and upregulated	EBV-LCLs, 5 Gy IR	[34]
miR-183	Recessive and downregulated	EBV-LCLs, 5 Gy IR	[34]
miR-96	Recessive and downregulated	EBV-LCLs, 5 Gy IR	[34]
miR-10a	Dominant and upregulated	EBV-LCLs, 5 Gy IR	[34]
miR-373	Dominant and upregulated	EBV-LCLs, 5 Gy IR	[34]
miR-199a-1	Dominant and downregulated	EBV-LCLs, 5 Gy IR	[34]
miR-218	Dominant and downregulated	EBV-LCLs, 5 Gy IR	[34]
miR-100	Dominant and downregulated	EBV-LCLs, 5 Gy IR	[34]
miR-125b	Dominant and downregulated	EBV-LCLs, 5 Gy IR	[34]
miR-99a	Dominant and downregulated	EBV-LCLs, 5 Gy IR	[34]
miR-34a-5p	Upregulation occurred with time in both A-T and controls but with no significant difference between them.	Primary T-lymphocytes, 2 Gy IR	[30]
miR-182-5p	Upregulation occurred with time in both A-T and controls but with no significant difference between them.	Primary T-lymphocytes, 2 Gy IR	[30]
miR-135a-5p	Upregulated	ATM⁻/⁻ vs. WT EBV-LCLs, 0.05 Gy & 0.5 Gy IR	[15]
miR-152-3p	Upregulated	ATM⁻/⁻ vs. WT EBV-LCLs, 0.05 Gy & 0.5 Gy IR	[15]
miR-223-3p	Upregulated	ATM⁻/⁻ vs. WT EBV-LCLs, 0.05 Gy & 0.5 Gy IR	[15]
miR-328-3p	Upregulated	ATM⁻/⁻ vs. WT EBV-LCLs, 0.05 Gy & 0.5 Gy IR	[15]
miR-424-5p	Upregulated	ATM⁻/⁻ vs. WT EBV-LCLs, 0.05 Gy & 0.5 Gy IR	[15]
miR-618	Upregulated	ATM⁻/⁻ vs. WT EBV-LCLs, 0.05 Gy & 0.5 Gy IR	[15]
miR-92a-1-5p	Upregulated	ATM⁻/⁻ vs. WT EBV-LCLs, 0.05 Gy & 0.5 Gy IR	[15]
miR-99a-5p	Upregulated	ATM⁻/⁻ vs. WT EBV-LCLs, 0.05 Gy & 0.5 Gy IR	[15]
miR-138-5p	Downregulated	ATM⁻/⁻ vs. WT EBV-LCLs, 0.05 Gy & 0.5 Gy IR	[15]
miR-141-3p	Downregulated	ATM⁻/⁻ vs. WT EBV-LCLs, 0.05 Gy & 0.5 Gy IR	[15]
miR-181d-5p	Downregulated	ATM⁻/⁻ vs. WT EBV-LCLs, 0.05 Gy & 0.5 Gy IR	[15]
miR-335-3p	Downregulated	ATM⁻/⁻ vs. WT EBV-LCLs, 0.05 Gy & 0.5 Gy IR	[15]
miR-501	Downregulated	ATM⁻/⁻ vs. WT EBV-LCLs, 0.05 Gy & 0.5 Gy IR	[15]
miR-497-5p	Downregulated	ATM⁻/⁻ vs. WT EBV-LCLs, 0.05 Gy & 0.5 Gy IR	[15]

### ATM-dependent LncRNA responses

Kabacik et al. measured selected ncRNAs in irradiated human T lymphocytes (one A-T patient vs. two healthy donors). In the first 2 h post irradiation, FAS-AS1 showed a significant rise in healthy controls as compared to A-T but later the difference diminished. It is assumed to be as a diagnostic marker just for < 2 h post radiation but not a very promising one. The other lncRNA TP53TG1 showed an uprise post radiation in all 3 samples but there was no difference between A-T and healthy controls as the expression was almost identical at the end [30]. In another study, Podralska et al. examined irradiated (1 Gy) lymphoblastoid cell lines from four A-T patients (ATM-null) and four healthy donors. Two timepoints were observed as of 1 h and 8 h, where 10 lncRNAs induced in healthy controls but not even one in A-T samples after 1 h. After 8 h post irradiation, 149 lncRNAs were induced in healthy controls whereas just 3 lncRNAs in A-T. Nine of the control lncRNAs were further validated by RT-qPCR. Three of them were induced at 1 h (i.e., LINC-SPTLC1-3, LINC-IER5-2, TNFRSF10B-AS-1), four at 8 h (i.e., LINC-TERB2-4, LINC-MYC-15, LINC-MYC-12, LINC-ZCCHC13-8), and two in both timeframes (i.e., XPC-AS-1, LINC-GPR157-1). The ATM kinase inhibitor KU-60,019 was used to check the ATM dependency of these lncRNAs. The induction was a weaker post inhibition, confirming ATM dependence. However, three lncRNAs (i.e., LINC-ZSCAN20-1, LINC-CD58-1, LINC-CBWD5-1*)* were induced exclusively in A-T lines, suggesting dysregulated pattern unique to ATM deficiency. Thus, the study identified that the functional ATM is required for most of the lncRNAs induction and provides a clear roadmap for future mechanistic investigation [17].

**Table 3:** List of LncRNAs in ataxia telangiectasia

Non-coding RNA	Expression pattern	Subjects/model	References
LINC-SPTLC1-3	Induced at 1 h only in controls; not in A-T	EBV-LCLs, 1 Gy IR	[17]
LINC-IER5-2	Induced at 1 h only in controls; not in A-T	EBV-LCLs, 1 Gy IR	[17]
TNFRSF10B-AS-1	Induced at 1 h only in controls; not in A-T	EBV-LCLs, 1 Gy IR	[17]
LINC-TERB2-4	Induced at 8 h only in controls; not in A-T	EBV-LCLs, 1 Gy IR	[17]
LINC-MYC-15	Induced at 8 h only in controls; not in A-T	EBV-LCLs, 1 Gy IR	[17]
LINC-MYC-12	Induced at 8 h only in controls; not in A-T	EBV-LCLs, 1 Gy IR	[17]
LINC-ZCCHC13-8	Induced at 8 h only in controls; not in A-T	EBV-LCLs, 1 Gy IR	[17]
XPC-AS-1	Induced at both 1 h and 8 h only in controls; not in A-T	EBV-LCLs, 1 Gy IR	[17]
LINC-GPR157-1	Induced at both 1 h and 8 h only in controls; not in A-T	EBV-LCLs, 1 Gy IR	[17]
LINC-ZSCAN20-1	Induced at 8 h only in A-T not in controls	EBV-LCLs, 1 Gy IR	[17]
LINC-CD58-1	Induced at 8 h only in A-T not in controls	EBV-LCLs, 1 Gy IR	[17]
LINC-CBWD5-1	Induced at 8 h only in A-T not in controls	EBV-LCLs, 1 Gy IR	[17]
FAS-AS1	Upregulation occurs with time but only in early hours post exposure, later no significant difference between A-T and controls	Primary T-lymphocytes, 2 Gy IR	[30]
TP53TG1	Upregulation occurs with time but not a significant difference between A-T and controls	Primary T-lymphocytes, 2 Gy IR	[30]

**Fig. 2:**
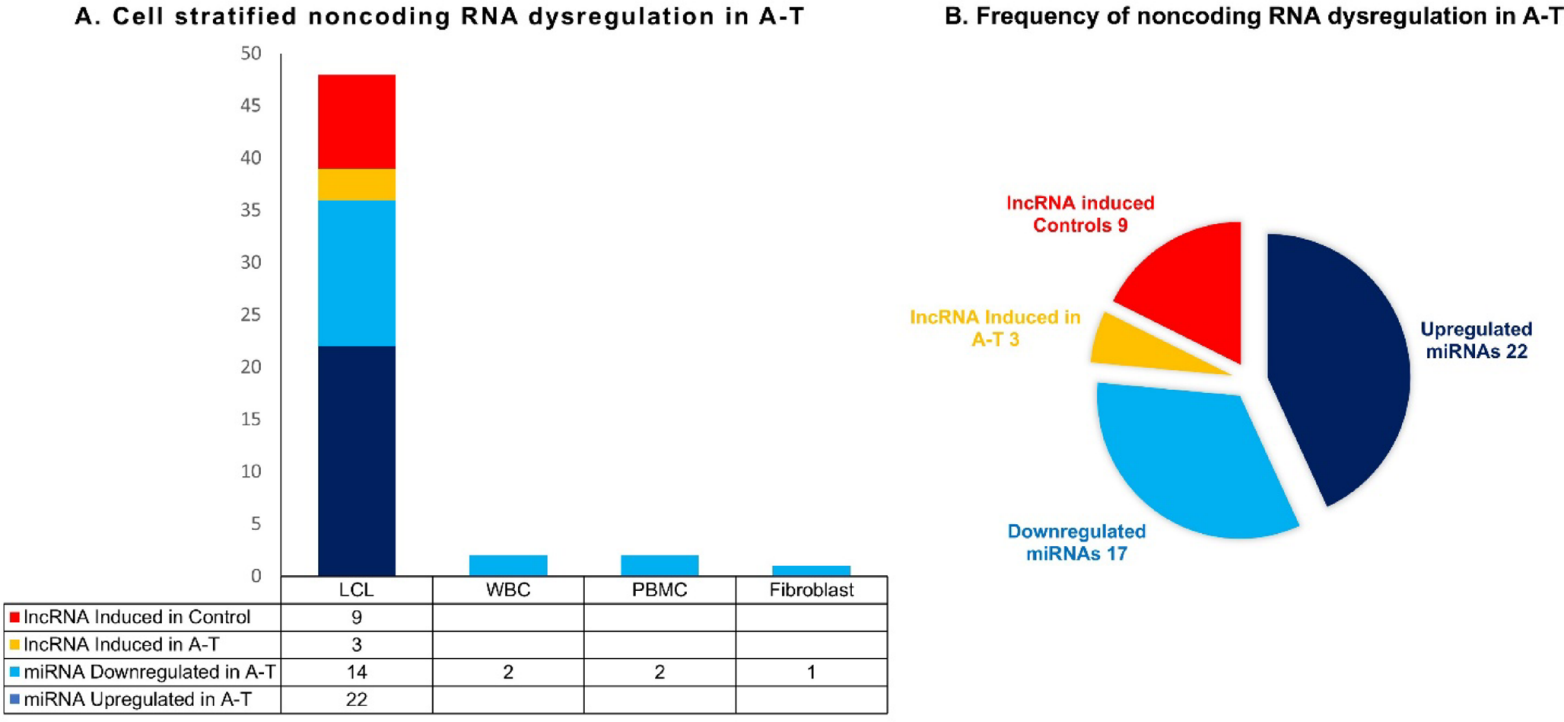
Cell-type stratification and overall burden of ncRNA dysregulation in A-T A: Stacked-column chart illustrating numbers of dysregulated ncRNAs detected in lymphoblastoid cell lines (LCL), white blood cells (WBC), peripheral-blood mononuclear cells (PBMC) and skin fibroblasts from A-T patients. LCLs harbor the greatest ncRNA imbalance, whereas primary blood and fibroblast samples show restricted, cell-specific profiles. B: Pie-chart summarizing the frequency of each category across all samples. (Made in Powerpoint)

**Fig. 3:**
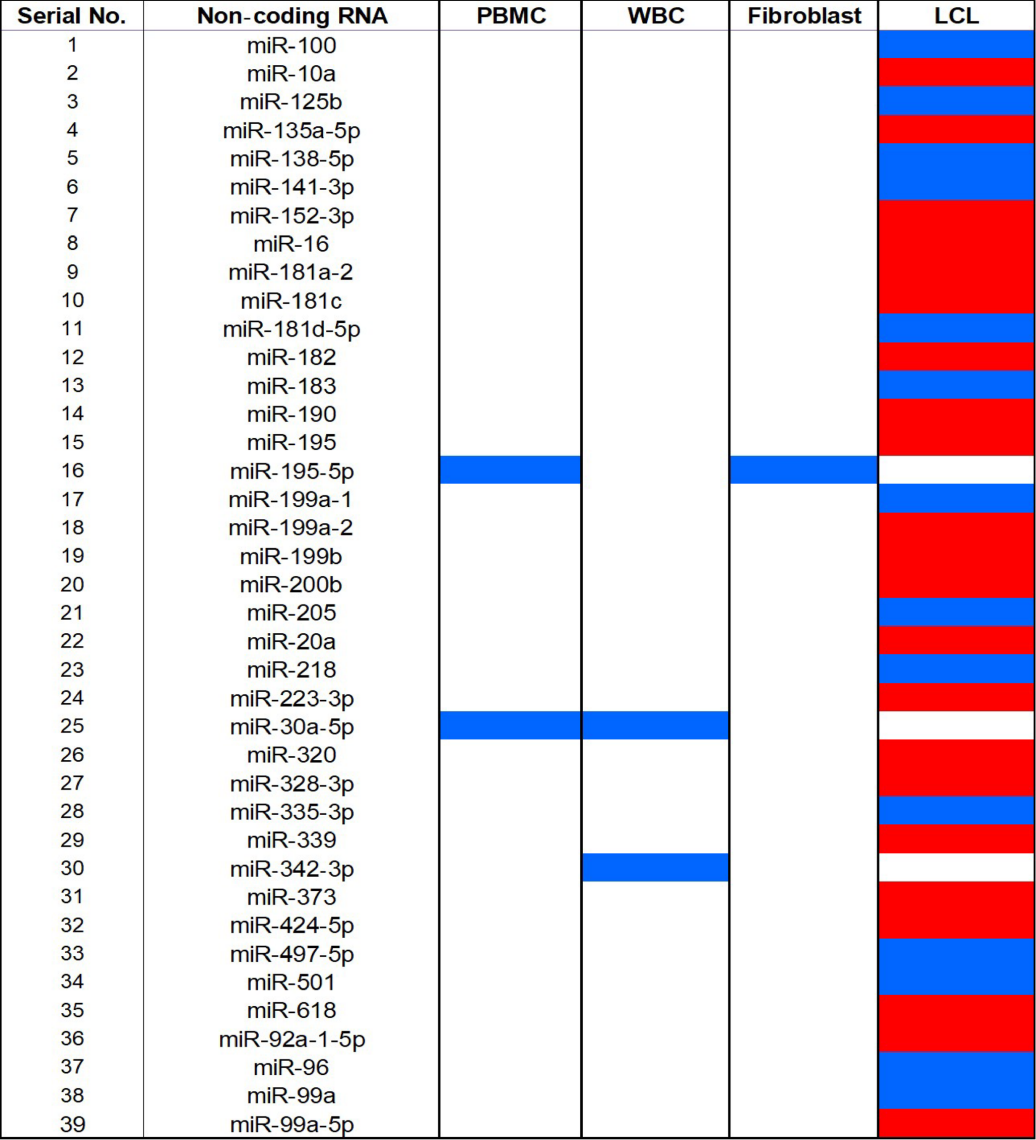
Heat-map overview of individual miRNA changes across A-T-derived cell types Matrix lists 39 microRNAs (rows) versus PBMC, WBC, fibroblast and LCL samples (columns). Colored cells indicate validated dysregulation: red shows upregulation or induction and blue shows downregulation or non-induction, and white means not measured. The LCL column displays the densest alteration pattern, with alternating red/blue blocks highlighting reciprocal regulation (e.g. miR-125b up, miR-138-5p down). In contrast, primary cells exhibit fewer but distinct signatures (e.g. miR-195-5p down in PBMC and fibroblasts). (Made in Excel)

Collectively in A-T, ATM deficiency plays a key role in ncRNAs expression pattern from baseline expression downregulation to distinct irradiation responsive signatures. The implications of these findings and ATM dependent role of other ncRNAs in context to other diseases are explored in the discussion section. The frequency and cell stratified dysregulation of these ncRNAs are shown in Figs. 2, 3.

## Discussion

We mapped out all five studies that examined the role of ncRNAs in Ataxia Telangiectasia. There were 5 eligible studies in which three studies checked miRNAs [15, 16, 34], one examined both miRNAs and lncRNAs [30] and one observed lncRNAs [17] only. They all provide the entire landscape of A-T-specific ncRNAs from inception to date from baseline levels to response to irradiation in blood and cell lines respectively. Circular RNA is still an unexplored avenue in A-T. Cirillo et al. examined miRNAs in PBMCs, fibroblasts and then validated them in WBCs. They found miR-195-5p, miR-30a-5p and miR-342-3p downregulated in different settings as described in results Sect [16]. Three other studies on miRNAs examined them under radiation stress. Bryant et al. gave a low dose irradiation on ATM-null lymphoblastoid cell lines (LCLs) which showed bidirectional regulation of miRNAs where eight miRNAs were up while six were downregulated relative to healthy control [15]. However, Kabacik et al. showed an opposite phenomenon where miR-34a-5p and miR-182-5p spiked before and after irradiation but the increase was largely ATM-independent as there was no difference between A-T and controls. Similar trend was observed in lncRNAs but FAS-AS1 was an exception post irradiation in early hours where they observed a difference between A-T and controls which diminished with time after 2 h [30]. Another study by Smirnov and colleagues distinguished miRNAs based on recessive vs. dominant IR-responsive miRNAs in EBV-transformed LCLs. They observed a similar bidirectional dysregulation pattern in multiple miRNAs both in recessive and dominant categories [34]. Irradiated LCLs from four patients and healthy controls demonstrated that 149 lncRNAs are ATM-dependent and only induced in WT; however, three others were uniquely induced in A-T cells only [17]. Although the circular-RNA layer remains less explored in the A-T context, individual mechanistic studies, in context of some other diseases, already found circATM in abdominal aortic aneurysms as modulators of oxidative stress and circ_0008657 when binds with miR-203-a-3p activates ATM to regulate the DNA-damage signaling [31, 35]. A natural knockout of ATM is present in A-T, yet only five primary studies have evaluated the behavior of ncRNAs in A-T. To assess the relationship of ncRNAs and ATM, many other studies were examined mechanistically in context of other diseases. We mapped different dimensions to assess the relationship between ATM and ncRNAs.

Multiple studies reported that some specific miRNAs bind to the 3′ untranslated region (3′-UTR) of *ATM* mRNA. This binding results in the lower expression of ATM halting the DNA damage response. A study reported that an oncogene *N-MYC* upregulates miR-421, which then binds to the *ATM* 3′-UTR. Over-expression of miR-421 in neuroblastoma or HeLa cells reduces ATM protein, resulted in S-phase cell cycle checkpoint changes and an increased sensitivity to IR resulting in creating a cellular phenotype similar to cells derived from A-T patients [20]. Similarly, Song et al. found that miR-18a directly targets the *ATM* 3′-UTR in breast cancer cell lines. The upregulation of miR-18a causes *ATM* downregulation in return reduced the DNA damage repair ability and the efficiency of homologous recombination-based DNA repair (HRR) and sensitized cells to IR treatment [21].

Some miRNAs of same family showed opposite activity in A-T and cancer. Bisso et al. observed miR-181a and miR-181b as a negative regulator of the DNA damage response in breast cancer. They reported that miR-181a and miR-181b overexpression in more aggressive breast cancers correlates inversely with ATM levels [36]. In leiomyosarcoma, miR-181b overexpression using mimic decreased *ATM* expression [37]. Similar to above studies that observed this inverse correlation in cancer, many miRNAs of same miR-181 family i.e., miR-181a-2 and miR-181c showed a recessive upregulation radiation response in A-T patient as compared to control [34] which may be due similar inverse correlation. In contrast to the above studies where *ATM* was mostly downregulated, Bryant et al. revealed a partially inverted pattern of the same miR-181 family in A-T. They reported that miR-181d-5p was significantly downregulated after low dose 0.05–0.5 Gy irradiation [15]. It is also reported as a negative regulator of cell cycle progression in uveal melanoma [38]. In contrast, cancer cells with partial ATM loss [36–38] or A-T cells under high dose irradiation of 5 Gy [34] often over-express the miRNA-181 family to further weaken the immune context. The miR-181d-5p could also change expression pattern if studied under high dose irradiation like other miRNA family members. The opposite behavior from similar family miRNA members could also occur as some A-T cells lacking ATM chronically might shed some miRNAs that suppress ATM, perhaps as a compensatory attempt to save any remaining DDR activity or cell cycle progression.

**Fig. 4:**
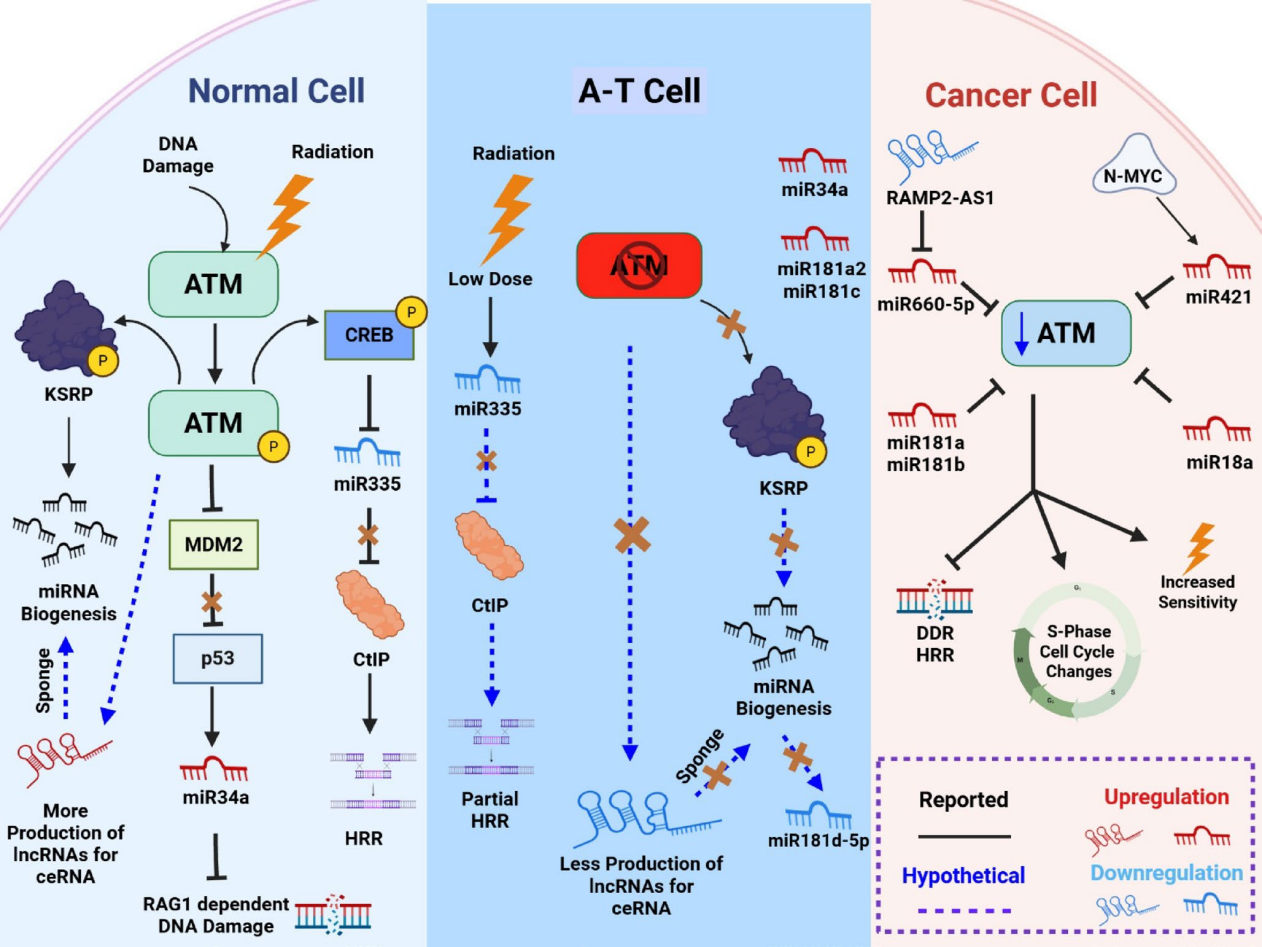
Schematic ATM–ncRNA crosstalk in normal, ataxia-telangiectasia (A-T) and cancer cells. ATM activation in normal cells phosphorylates KSRP, CREB and inhibits MDM2, boosting miRNA biogenesis, homologous recombination repair and inhibition of RAG1 dependent DNA damage respectively. In A-T, loss of ATM blocks these phosphorylation events affecting miRNA processing, reduced lncRNA for sponging, and CtIP-mediated repair. Cancer cells often repress ATM through oncogenic or lncRNA-driven miRNAs (i.e., miR-181a/b, miR-421, miR-660-5p, miR-18a), causing S-phase stress and hypersensitivity to radiation. Solid lines denote reported links; blue dashed arrows indicate hypothetical ones; red and blue RNA icons show up or downregulation, respectively. (Created in BioRender. Iqbal, M. (2025) https://BioRender.com/pc8jndz)

Early mechanistic studies revealed that *ATM* is not just a downstream target of miRNAs but also an important regulator of DNA damage-induced non-coding RNAs. In a key study, Zhang et al. reported that nearly one-fourth of miRNAs are notably upregulated following DNA damage, whereas the absence of *ATM* completely halts this induction. KH-type splicing regulatory protein (KSRP) is a key player that translates DNA damage signaling to miRNA biogenesis. ATM directly phosphorylates KSRP, enhancing its interaction with pri-miRNAs and boosting its function in miRNA processing [39]. Cells deficient in ATM failed to exhibit this characteristic surge in miRNA expression, clearly positioning ATM as an upstream regulator in miRNA biogenesis. The pattern of downregulation of certain miRNAs observed in A-T studies may be linked to their dependence on KSRP. Bryant et al. observed a noisy output in A-T LCLs where eight miRNAs upregulated after irradiation, but six others were simultaneously repressed [15]. It can be hypothesized that the downregulation of ATM causes loss of KSRP mediated processing of pri-miRNAs which results in downregulation of some miRNAs. In other A-T studies [16, 34], there may be a chance of similar ATM dependent regulation of KSRP and pri-miRNAs processing network which leads to an ncRNA deficiency. A hypothetical link between downregulation of these miRNAs and KSRP mediated biogenesis is shown in Fig. 4 using a dotted line. More studies are needed to explore the link between downregulated miRNAs and KSRP to further strengthen this hypothesis.

The competing endogenous RNA (ceRNA) hypothesis suggests that lncRNAs, circRNAs, and pseudogene transcripts can bind and sequester miRNAs, thereby alleviating repression on shared mRNA targets [40]. In breast-cancer cells, hypoxia suppresses the lncRNA RAMP2-AS1. Because RAMP2-AS1 normally sponges miR-660-5p, its downregulation frees miR-660-5p to bind the 3′-UTR of *ATM* mRNA, thereby restoring the miRNA’s inhibitory effect on *ATM* and resulting in lower ATM protein levels. This RAMP2-AS1/miR-660-5p/ATM axis represents a potential hypoxia-associated regulatory mechanism with prognostic significance in breast cancer [41]. Podralska et al. identified 149 ATM-dependent lncRNAs (nine were validated) induced by IR in control lymphoblastoid cells, which was ATM dependent but absent in ATM-null A-T cells, while only three were unique to A-T lines [17]. We hypothesized that in ATM-deficient A-T cells, the typical surge of ATM-inhibiting miRNAs fails to occur, which might eliminate the need for lncRNA sponges that normally sequester them. However, functional ATM in healthy cells first promotes the production of KSRP mediated miRNAs. Some of the lncRNAs might be produced to sponge the excess miRNAs that repress or directly target ATM expression, thereby protecting ATM protein levels. This cellular mechanism also clarifies why miRNA patterns observed in ATM-low cancer tumor cells do not directly correlate with complete ATM-null A-T cells. Collectively, these findings establish ceRNA networks as a third regulatory level complementing transcriptional and post-translational controls, offering potential therapeutic opportunities for Ataxia Telangiectasia.

Additionally, in the context of DNA damage response, ATM regulates p53 when senses a break by phosphorylating it on Ser-15. It then blocks MDM2 to activate the transcription of p53 [42]. Another study reported that DNA damage triggers ATM activation leading to stabilizing p53 which then activates the p53 effectors, including miR-34a inhibiting RAG1 dependent DNA damage. This directly shows the relationship of ATM mediated miR34a regulation by p53 in response to DNA damage [43]. The upregulation of the same miR-34a occurred with time in both A-T and controls but with no significant difference between them [30]. We can speculate that miR-34a has both ATM-dependent [43] and ATM-bypass routes [30], so its kinetics look similar in A-T and controls. Sometimes deregulation of miRNAs might happen to preserve p53 mediated DDR activity in ATM null A-T cells. Another coherent ATM-miR-335 axis emerges when Martin et al. reported that the radiation activated ATM phosphorylates CREB, reducing its binding at the miR-335 promoter and lowering miR-335 levels. This derepresses C-terminal binding protein-interacting protein (CtIP) to promote homologous recombination repair [44]. In contrast, EBV-transformed lymphoblastoid cells exposed only to low-dose IR showed constitutively lower miR-335-3p in ATM-null lines than in wild-type controls [15]. It implies an additional basal control layer such as defective KSRP processing is present that might depresses miR-335 when ATM is absent to save some DDR activity which otherwise could be lost leading to repression of HRR. Findings from LCLs should therefore be considered model-dependent and not directly extrapolated to patient tissues. Their divergence from PBMCs or fibroblasts likely arises from immortalization and lineage context, which limits their physiological relevance. Future studies profiling ncRNAs in neuronal and other clinically affected tissues will be critical to resolve these discrepancies. An important future perspective will be to validate whether specific miRNA profiles can stratify the clinical phenotype of A-T, particularly in relation to cancer susceptibility. Prospective cohort studies that link circulating or tissue-specific miRNA expression to cancer incidence in A-T patients are needed to establish their biomarker potential.

## Therapeutic targeting of miRNA-ATM regulatory axis

Research on ATM can be shifted from theoretical science to applied approach by attempting to restore ATM function in A-T cells, either by blocking miRNAs that suppress ATM or by supplementing those that support its expression. Inhibitors of miR-421 in neuroblastoma and HeLa cells led to an increased expression of ATM protein. With ATM restored, S-phase DNA damage checkpoints resumed normal function thus enhancing sensitivity to radiations [20]. The inhibitors of miR-18a and miR181a, and miR-181b in breast cancer cell lines improved DNA repair after radiation as shown by fewer γ-H2AX foci [21, 36]. In contrast, A-T cells that completely lack functional ATM protein have persistently low levels of specific miRNAs e.g., miR-181d-5p [15]. Therapeutically, supplementing miR-181d-5p in A-T cells (where it is deficient) while inhibiting over expressed miR-181a/b (and related family members) in cancers that still retain some ATM activity is the best possible approach because the two settings show opposite miR-181 family dysregulation and thus require opposite interventions.

An additional approach is to use ceRNA network in which ncRNAs (lncRNAs or circRNAs) trap specific regulatory miRNAs, a concept described as ‘sponging’. A study by Podralska et al. identified 149 lncRNAs that are upregulated when ATM is functional [17]. One of them, LINC-TERB2-4, can be experimentally tested to check if it performs this sponge like function by sequestering miRNAs that inhibit ATM expression. This idea is supported by experimental evidence in breast cancer cells, where the lncRNA RAMP2-AS1, was shown to bind and inhibit miR-660-5p, allowing increased ATM production [41]. Circular RNAs are more resistant to degradation by nucleases. One such naturally occurring RNA called endogenous circATM modulates oxidative stress in smooth muscle cells [31]. These circular RNAs are still an unexplored dimension in A-T, but if studied, they may act as effective therapeutic based signatures. Clinical success of RNA drugs like Nusinersen [45] and Patisiran [46] establishes a viable regulatory pathway for advancing ncRNA based therapies in A-T, despite the challenges of unwanted side effects, consistent delivery to cerebellum and GMP manufacturing.

While ncRNAs represent attractive targets, the concept of manipulating miRNA levels as a therapeutic strategy in A-T remains premature. The currently available studies are limited in number and lack functional validation, so interventional approaches should for now be considered hypothesis-generating rather than actionable. Another important consideration for any RNA-based intervention is safety. miRNA mimics or inhibitors can have off-target effects through unintended binding to transcripts with partial seed sequence complementarity, or by saturating components of the RNA-induced silencing complex. Moreover, therapeutic benefit in A-T would likely require cell-type specific delivery, as systemic modulation of miRNAs may disrupt normal regulatory networks in unaffected tissues. Approaches such as targeted nanoparticles, viral vectors with restricted tropism, or chemically modified antisense oligonucleotides may help achieve the necessary specificity and safety profile.

## Gaps and limitations

Although these five A-T studies provide the first comprehensive picture of how ncRNAs are dysregulated in A-T, there are still major ncRNAs like circRNA that remain unexplored. Even though there is evidence that lncRNAs can sequester miRNAs that would otherwise target and suppress ATM [41], no study has investigated whether this mechanism occurs in A-T with circRNAs, snoRNAs, piRNAs or tRNAs derived fragments. All five studies have used PBMCs, LCLs or fibroblasts but none have examined the cell types and tissues most severely affected in A-T, such as neuronal, muscular, or epithelial cells. To overcome these limitations, future studies will require advanced tools and models, including longitudinal multiomics cohorts, CRISPR-edited models, genome wide CRISPR-interference rescue screens and in vivo delivery of ncRNA mimics or anti-miRs in ATM-/- mice.

This study is strengthened by a multi-database search strategy, independent dual screening, and transparent data extraction, all of which reduce the risk of bias and ensure inclusion and synthesis of both baseline and stress-response studies. Importantly, this study does not rely solely on data from A-T. It also draws on mechanistic studies for discussion from other diseases or systems which help support the idea that certain biological mechanisms are consistent across different contexts. The number of relevant studies is very small (*n* = 5), and most of them are based on lymphoblastoid cell lines under irradiation, which may not accurately reflect the biology of tissues most affected in A-T, such as brain or immune system. As this is a scoping review, we did not assess the risk of bias in original studies; therefore, any methodological limitations in those studies were not formally identified or quantified. Small studies or those that did not demonstrate a strong impact of ncRNA in A-T, may have remained unpublished, leading to a potential publication bias. Only English language studies were included. Additionally, we did not perform a comprehensive search of non-traditional sources such as conference proceedings or student theses.

## Conclusions & future perspective

Till now, only five studies have been published primarily using PBMCs, LCLs and fibroblast models. Loss of ATM leads to reduction in a specific subgroup of miRNAs whose biogenesis may depend on RNA binding protein KSRP which might also block DNA damage repair related 149 lncRNAs and disrupt gene regulation networks like ceRNA and p53 in a unique way not observed in cancers that only exhibit low ATM expressions. Despite some progress of circRNA and ATM link in other diseases, important gaps still exist in our understanding of circRNAs in A-T. The most affected tissues (e.g., neuronal, muscular or epithelial) have not yet been profiled for ncRNA expression. Furthermore, only a small number of dysregulated ncRNAs have been experimentally tested to see if they influence ATM, p53, or DNA repair mechanisms. In future, early research priorities should include intermediate dose stress models, comprehensive total and small RNA sequencing, and CRISPR-based rescue or knockout of candidate miRNA-lncRNA pairs. To transform ncRNA research into effective treatments, we need to test it in realistic models, such as animal models or patient derived organoids as well as validate them. Therefore, the immediate priority should be rigorous biomarker validation, while therapeutic applications should await stronger experimental and translational evidence. This will help validate which non-coding RNAs are useful as disease markers or therapeutic targets particularly for approaches aimed at compensating for the functional loss caused by *ATM* gene dysregulation. The projects should be built as a comprehensive analytical pipeline that integrates total RNA sequencing and microarrays analysis in ATM deficient cells to investigate drug induced transcriptional changes, predict functional miRNA biomarkers, and check if they restore normal function in A-T cells carrying distinct *ATM* mutations. The goal is to deepen understanding of gene regulatory mechanisms in A-T and to advance the development of ncRNA-based diagnostic and therapeutic strategies.

## Supplementary Information

Below is the link to the electronic supplementary material.

Supplementary Material 1

Supplementary Material 2

## Data Availability

No datasets were generated or analysed during the current study.

## References

[1] Amirifar P, Ranjouri MR, Yazdani R, Abolhassani H, Aghamohammadi A (2019) Ataxia-telangiectasia: a review of clinical features and molecular pathology. Pediatr Allergy Immunol 30(3):277–288 30685876 10.1111/pai.13020

[2] Soukupova J, Pohlreich P, Seemanova E (2011) Characterisation of ATM mutations in Slavic ataxia telangiectasia patients. Neuromolecular Med 13:204–211 21833744 10.1007/s12017-011-8152-z

[3] Hummel JM, Thorland EC, Lim MS (2010) Hodgkin lymphoma in a young child contributing to a diagnosis of ataxia telangiectasia: review of the literature. J Hematop 3:69–76

[4] Magnarelli A, Liu Q, Wang F, Peng XP, Wright J, Oak N, Natale V, Rothblum-Oviatt C, Lefton-Greif MA, McGrath-Morrow S, Crawford TO, Ehrhardt MJ, Lederman HM, Sharma R (2025) Prevalence and outcomes of cancer and treatment-associated toxicities for patients with ataxia telangiectasia. J Allergy Clin Immunol 155(2):640–649 39521281 10.1016/j.jaci.2024.10.023 PMC11915532

[5] Tiet MY, Guțu BI, Springall-Jeggo P, Coman D, Willemsen M, Van Os N, Doria M, Donath H, Schubert R, Dineen RA, Biagiotti S, Prayle AP, Group ATBW, Hensiek AE, Horvath R (2025) Biomarkers in Ataxia-telangiectasia: a systematic review. J Neurol 272(2):110 39812834 10.1007/s00415-024-12766-7 PMC11735505

[6] Choy KR, Watters DJ (2018) Neurodegeneration in ataxia-telangiectasia: multiple roles of ATM kinase in cellular homeostasis. Dev Dyn 247:(1):33–46 28543935 10.1002/dvdy.24522

[7] Chan SF, Sances S, Brill LM, Okamoto S-i, Zaidi R, McKercher SR, Akhtar MW, Nakanishi N, Lipton SA (2014) Atm-dependent phosphorylation of MEF2D promotes neuronal survival after DNA damage. J Neurosci 34(13):4640–4653 24672010 10.1523/JNEUROSCI.2510-12.2014 PMC3965787

[8] Huff LA, Yan S, Clemens MG (2021) Mechanisms of ataxia telangiectasia mutated (ATM) control in the DNA damage response to oxidative stress, epigenetic regulation, and persistent innate immune suppression following sepsis. Antioxid (Basel) 10(7):1146 10.3390/antiox10071146 PMC8301080 34356379

[9] Burma S, Chen BP, Murphy M, Kurimasa A, Chen DJ (2001) ATM phosphorylates histone H2AX in response to DNA double-strand breaks. J Biol Chem 276:(45):42462–42467 11571274 10.1074/jbc.C100466200

[10] Ratnaparkhe M, Hlevnjak M, Kolb T, Jauch A, Maass K, Devens F, Rode A, Hovestadt V, Korshunov A, Pastorczak A (2017) Genomic profiling of acute lymphoblastic leukemia in ataxia telangiectasia patients reveals tight link between ATM mutations and chromothripsis. Leukemia 31(10):2048–2056 28196983 10.1038/leu.2017.55

[11] McKinnon PJ (2012) ATM and the molecular pathogenesis of ataxia telangiectasia. Annu Rev Pathol Mech Dis 7:(1):303–321 10.1146/annurev-pathol-011811-132509 22035194

[12] Zielen S, Crawford T, Benatti L, Magnani M, Kieslich M, Ryan M, Meyts I, Gulati S, Borgohain R, Yadav R (2024) Safety and efficacy of intra-erythrocyte dexamethasone sodium phosphate in children with ataxia telangiectasia (ATTeST): a multicentre, randomised, double-blind, placebo-controlled phase 3 trial. Lancet Neurol 23(9):871–882 39152028 10.1016/S1474-4422(24)00220-5

[13] Broccoletti T, Giudice ED, Cirillo E, Vigliano I, Giardino G, Ginocchio V, Bruscoli S, Riccardi C, Pignata C (2011) Efficacy of very-low‐dose betamethasone on neurological symptoms in ataxia‐telangiectasia. Eur J Neurol 18(4):564–570 20840352 10.1111/j.1468-1331.2010.03203.x

[14] Zannolli R, Buoni S, Betti G, Salvucci S, Plebani A, Soresina A, Pietrogrande MC, Martino S, Leuzzi V, Finocchi A (2012) A randomized trial of oral betamethasone to reduce ataxia symptoms in ataxia telangiectasia. Mov Disord 27(10):1312–1316 22927201 10.1002/mds.25126

[15] Bryant J, White L, Coen N, Shields L, McClean B, Meade AD, Lyng FM, Howe O (2020) Microrna analysis of ATM-deficient cells indicate PTEN and CCDN1 as potential biomarkers of radiation response. Radiat Res 193(6):520–530 32216710 10.1667/RR15462.1

[16] Cirillo E, Tarallo A, Toriello E, Carissimo A, Giardino G, Rosa AD, Damiano C, Soresina A, Badolato R, Dellepiane RM (2024) Microrna dysregulation in ataxia telangiectasia. Front Immunol 15:1444130 39224604 10.3389/fimmu.2024.1444130 PMC11366618

[17] Podralska M, Sajek MP, Bielicka A, Żurawek M, Ziółkowska-Suchanek I, Iżykowska K, Kolenda T, Kazimierska M, Kasprzyk ME, Sura W (2024) Identification of ATM-dependent long non-coding RNAs induced in response to DNA damage. DNA Repair 135:103648 38382170 10.1016/j.dnarep.2024.103648

[18] Zhao K, Wang X, Xue X, Li L, Hu Y (2020) A long noncoding RNA sensitizes genotoxic treatment by attenuating ATM activation and homologous recombination repair in cancers. PLoS Biol 18(3):e3000666 32203529 10.1371/journal.pbio.3000666 PMC7138317

[19] Wan G, Liu Y, Han C, Zhang X, Lu X (2014) Noncoding RNAs in DNA repair and genome integrity. Antioxid Redox Signal 20(4):655–677 23879367 10.1089/ars.2013.5514 PMC3901350

[20] Hu H, Du L, Nagabayashi G, Seeger RC, Gatti RA (2010) ATM is down-regulated by N-Myc–regulated microrna-421. Proc Natl Acad Sci U S A 107(4):1506–1511 20080624 10.1073/pnas.0907763107 PMC2824372

[21] Song L, Lin C, Wu Z, Gong H, Zeng Y, Wu J, Li M, Li J (2011) MiR-18a impairs DNA damage response through downregulation of ataxia telangiectasia mutated (ATM) kinase. PLoS ONE 6(9):e25454 21980462 10.1371/journal.pone.0025454 PMC3181320

[22] Cannavicci A, Zhang Q, Kutryk MJ (2020) Non-coding RNAs and hereditary hemorrhagic telangiectasia. J Clin Med 9:(10):3333 33080889 10.3390/jcm9103333 PMC7603193

[23] Pirola L, Ciesielski O, Biesiekierska M, Balcerczyk A (2021) *Modulation of noncoding RNAs (ncRNAs) and their potential role as therapeutics*, in *Med. Epigenetics*. Elsevier, pp 721–740

[24] Mattick JS, Amaral PP, Carninci P, Carpenter S, Chang HY, Chen L-L, Chen R, Dean C, Dinger ME, Fitzgerald KA (2023) Long non-coding rnas: definitions, functions, challenges and recommendations. Nat Rev Mol Cell Biol 24(6):430–447 36596869 10.1038/s41580-022-00566-8 PMC10213152

[25] El-Ashmawy NE, Khedr EG, Darwish RT, Ibrahim AO (2024) Competing endogenous RNAs network and therapeutic implications: new horizons in disease research. Biochim. Biophys Acta Gene Regul Mech 1868(1):195073 10.1016/j.bbagrm.2024.195073 39631541

[26] Wu K, Bu F, Wu Y, Zhang G, Wang X, He S, Liu M-F, Chen R, Yuan H (2024) Exploring noncoding variants in genetic diseases: from detection to functional insights. J Genet Genomics 51:(2):111–132 38181897 10.1016/j.jgg.2024.01.001

[27] Bartel DP (2018) Metazoan micrornas cell. Cell 173(1):20–51 29570994 10.1016/j.cell.2018.03.006 PMC6091663

[28] Guiducci G, Stojic L (2021) Long noncoding RNAs at the crossroads of cell cycle and genome integrity. Trends Genet 37(6):528–546 33685661 10.1016/j.tig.2021.01.006

[29] Ruffo P, De Amicis F, Giardina E, Conforti FL (2023) Long-noncoding RNAs as epigenetic regulators in neurodegenerative diseases. Neural Regen Res 18(6):1243–1248 36453400 10.4103/1673-5374.358615 PMC9838156

[30] Kabacik S, Manning G, Raffy C, Bouffler S, Badie C (2015) Time, dose and ataxia telangiectasia mutated (ATM) status dependency of coding and noncoding RNA expression after ionizing radiation exposure. Radiat Res 183:(3):325–337 25738893 10.1667/RR13876.1

[31] Fasolo F, Winski G, Li Z, Wu Z, Winter H, Ritzer J, Glukha N, Roy J, Hultgren R, Pauli J (2023) The circular RNA ataxia telangiectasia mutated regulates oxidative stress in smooth muscle cells in expanding abdominal aortic aneurysms. Mol Ther 33:848–865 10.1016/j.omtn.2023.08.017 PMC10481153 37680984

[32] Tricco AC, Lillie E, Zarin W, O’Brien KK, Colquhoun H, Levac D, Moher D, Peters MDJ, Horsley T, Weeks L, Hempel S, Akl EA, Chang C, McGowan J, Stewart L, Hartling L, Aldcroft A, Wilson MG, Garritty C, Lewin S, Godfrey CM, Macdonald MT, Langlois EV, Soares-Weiser K, Moriarty J, Clifford T, Tunçalp Ö, Straus SE (2018) Prisma extension for scoping reviews (PRISMA-ScR): checklist and explanation. Ann Intern Med 169(7):467–473 30178033 10.7326/M18-0850

[33] Page MJ, McKenzie JE, Bossuyt PM, Boutron I, Hoffmann TC, Mulrow CD, Shamseer L, Tetzlaff JM, Akl EA, Brennan SE (2021) The PRISMA 2020 statement: an updated guideline for reporting systematic reviews. BMJ 372:71. 10.1136/bmj.n71 10.1136/bmj.n71 PMC8005924 33782057

[34] Smirnov DA, Cheung VG (2008) ATM gene mutations result in both recessive and dominant expression phenotypes of genes and MicroRNAs. Am J Hum Genet 83:(2):243–253 18674748 10.1016/j.ajhg.2008.07.003 PMC2495067

[35] Chen W, Chen Z, Jia Y, Guo Y, Zheng L, Yao S, Shao Y, Li M, Mao R, Jiang Y (2024) Circ_0008657 regulates lung DNA damage induced by hexavalent chromium through the miR-203a-3p/ATM axis. Environ Int 185:108515 38394914 10.1016/j.envint.2024.108515

[36] Bisso A, Faleschini M, Zampa F, Capaci V, Santa JD, Santarpia L, Piazza S, Cappelletti V, Daidone M, Agami R, G Del Sal (2013) Oncogenic miR-181a/b affect the DNA damage response in aggressive breast cancer. Cell Cycle 12:(11):1679–1687 23656790 10.4161/cc.24757 PMC3713126

[37] de Almeida BC, Dos Anjos LG, Kagohara LT, Al-Hendy A, Yang Q, Baracat EC, Coutinho-Camillo CM, Carvalho KC (2025) Could let-7f, miR-10b, miR-34a, miR-181b, and miR-181d be useful tools as a target therapy for. uterine leiomyosarcoma? Biomedicines 13:3 10.3390/biomedicines13030560 PMC11940384 40149537

[38] Zhang L, He X, Li F, Pan H, Huang X, Wen X, Zhang H, Li B, Ge S, Xu X (2018) The miR-181 family promotes cell cycle by targeting CTDSPL, a phosphatase-like tumor suppressor in uveal melanoma. J Exp Clin Cancer Res 37:1–13 29382357 10.1186/s13046-018-0679-5 PMC5791374

[39] Zhang X, Wan G, Berger FG, He X, Lu X (2011) The ATM kinase induces microRNA biogenesis in the DNA damage response. Mol Cell 41(4):371–383 21329876 10.1016/j.molcel.2011.01.020 PMC3114434

[40] Salmena L, Poliseno L, Tay Y, Kats L, Pandolfi PP (2011) A cerna hypothesis: the Rosetta stone of a hidden. RNA language? Cell 146(3):353–358 21802130 10.1016/j.cell.2011.07.014 PMC3235919

[41] Lou W, Xiao S, Lin K (2024) Identification of a hypoxia-suppressed LncRNA RAMP2-AS1 in breast cancer. Noncoding RNA Res 9:(3):782–795 38590436 10.1016/j.ncrna.2024.02.007 PMC10999373

[42] Lee J-H (2024) Targeting the ATM pathway in cancer: opportunities, challenges and personalized therapeutic strategies. Cancer Treat Rev 129:102808 39106770 10.1016/j.ctrv.2024.102808

[43] Ochodnicka-Mackovicova K, van Keimpema M, Spaargaren M, van Noesel CJM, Guikema JEJ (2024) DNA damage-induced p53 downregulates expression of RAG1 through a negative feedback loop involving miR-34a and FOXP1. J Biol Chem 300(12):107922 39454960 10.1016/j.jbc.2024.107922 PMC11625342

[44] Martin NT, Nakamura K, Davies R, Nahas SA, Brown C, Tunuguntla R, Gatti RA, Hu H (2013) ATM–dependent MiR-335 targets CtIP and modulates the DNA damage response. PLoS Genet 9(5):e1003505 23696749 10.1371/journal.pgen.1003505 PMC3656122

[45] Finkel RS, Mercuri E, Darras BT, Connolly AM, Kuntz NL, Kirschner J, Chiriboga CA, Saito K, Servais L, Tizzano E, Topaloglu H, Tulinius M, Montes J, Glanzman AM, Bishop K, Zhong ZJ, Gheuens S, Bennett CF, Schneider E, Farwell W, De Vivo DC (2017) Nusinersen versus sham control in infantile-onset spinal muscular atrophy. N Engl J Med 377(18):1723–1732 29091570 10.1056/NEJMoa1702752

[46] Adams D, Gonzalez-Duarte A, O’Riordan WD, Yang CC, Ueda M, Kristen AV, Tournev I, Schmidt HH, Coelho T, Berk JL, Lin KP, Vita G, Attarian S, Planté-Bordeneuve V, Mezei MM, Campistol JM, Buades J, Brannagan TH 3, Kim BJ, Oh J, Parman Y, Sekijima Y, Hawkins PN, Solomon SD, Polydefkis M, Dyck PJ, Gandhi PJ, Goyal S, Chen J, Strahs AL, Nochur SV, Sweetser MT, Garg PP, Vaishnaw AK, Gollob JA, Suhr OB (2018) Patisiran, an RNAi therapeutic, for hereditary transthyretin amyloidosis. N Engl J Med 379(1):11–21 29972753 10.1056/NEJMoa1716153

