# Supplementary material for: Mapping the non-coding RNA landscape in ataxia telangiectasia: a scoping review of ATM dependent miRNA and lncRNA dysregulation
Source: Mol Biol Rep. 2025 Oct 9;52(1):998. doi: 10.1007/s11033-025-11094-x (PMC12511216; doi:10.1007/s11033-025-11094-x)
Supplement: Supplementary file 2 — Supplementary Material 2 [file 11033_2025_11094_MOESM2_ESM.docx]

**PubMed**

micrornas OR rna, untranslated OR rna, circular OR rna, long noncoding OR rna, untranslated OR circulating noncoding rna OR noncoding rna AND ataxia telangiectasia

**Search Results: 662**

**
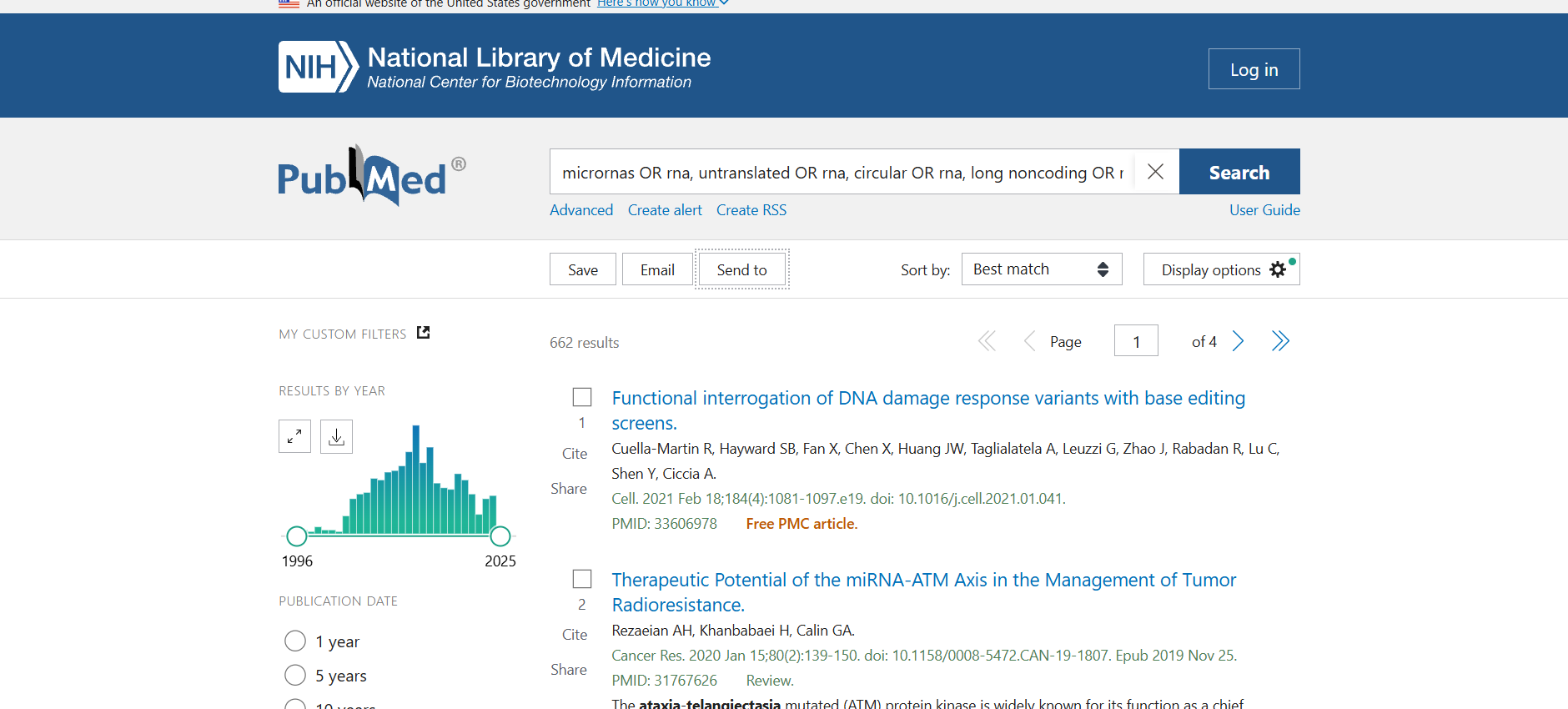
**

**Scopus**

micrornas OR rna, untranslated OR rna, circular OR rna, long noncoding OR rna, untranslated OR circulating noncoding rna OR noncoding rna AND ataxia telangiectasia

**Scopus Results: 605
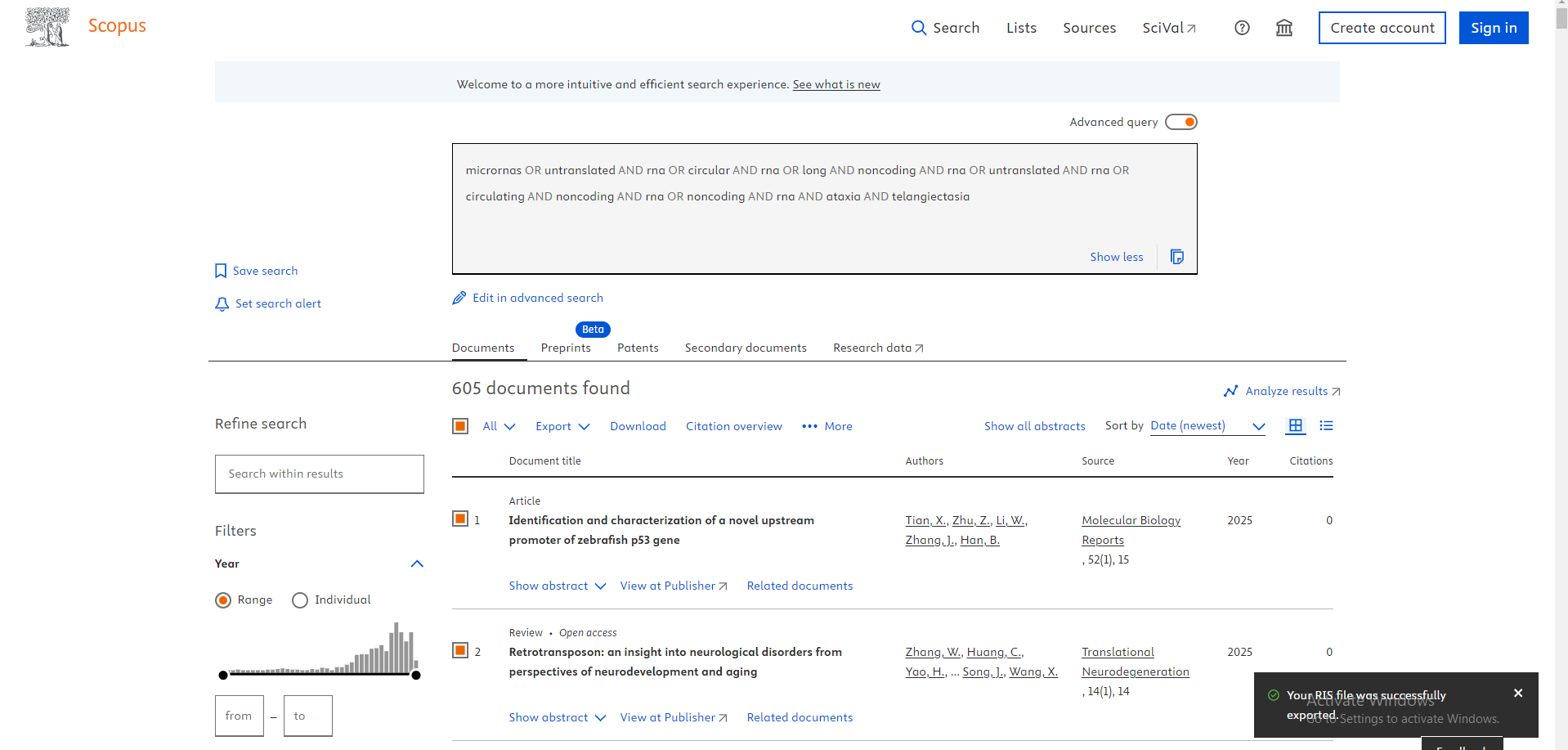
**

**Web of Science**

**Search Term:**(((((ALL=(micrornas)) OR ALL=(circular RNA)) OR ALL=(long noncoding rna)) OR ALL=(noncoding RNA)) OR ALL=(untranslated RNA)) AND ALL=(ataxia telangiectasia)

**Results:** 79

**
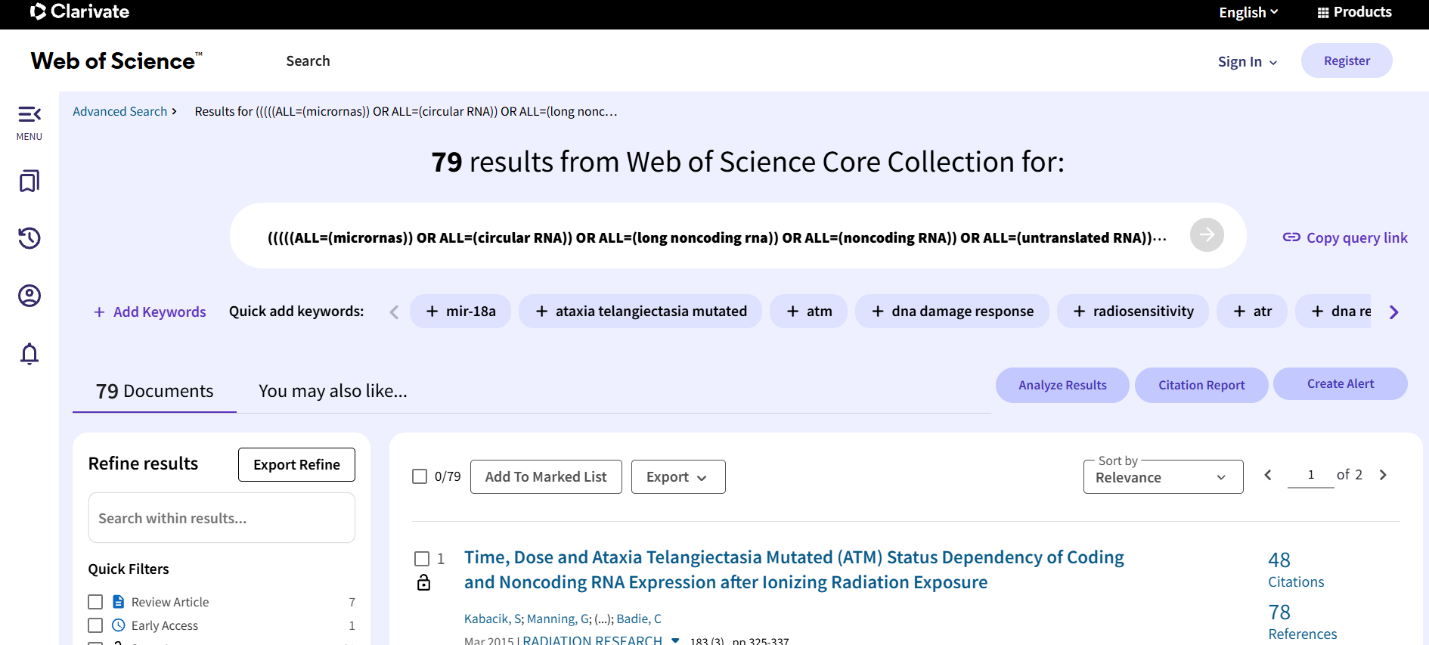
**

**Embase**

**Search Term:** micrornas OR rna, untranslated OR rna, circular OR rna, long noncoding OR rna, untranslated OR circulating noncoding rna OR noncoding rna AND ataxia telangiectasia

**Results:** 734

**
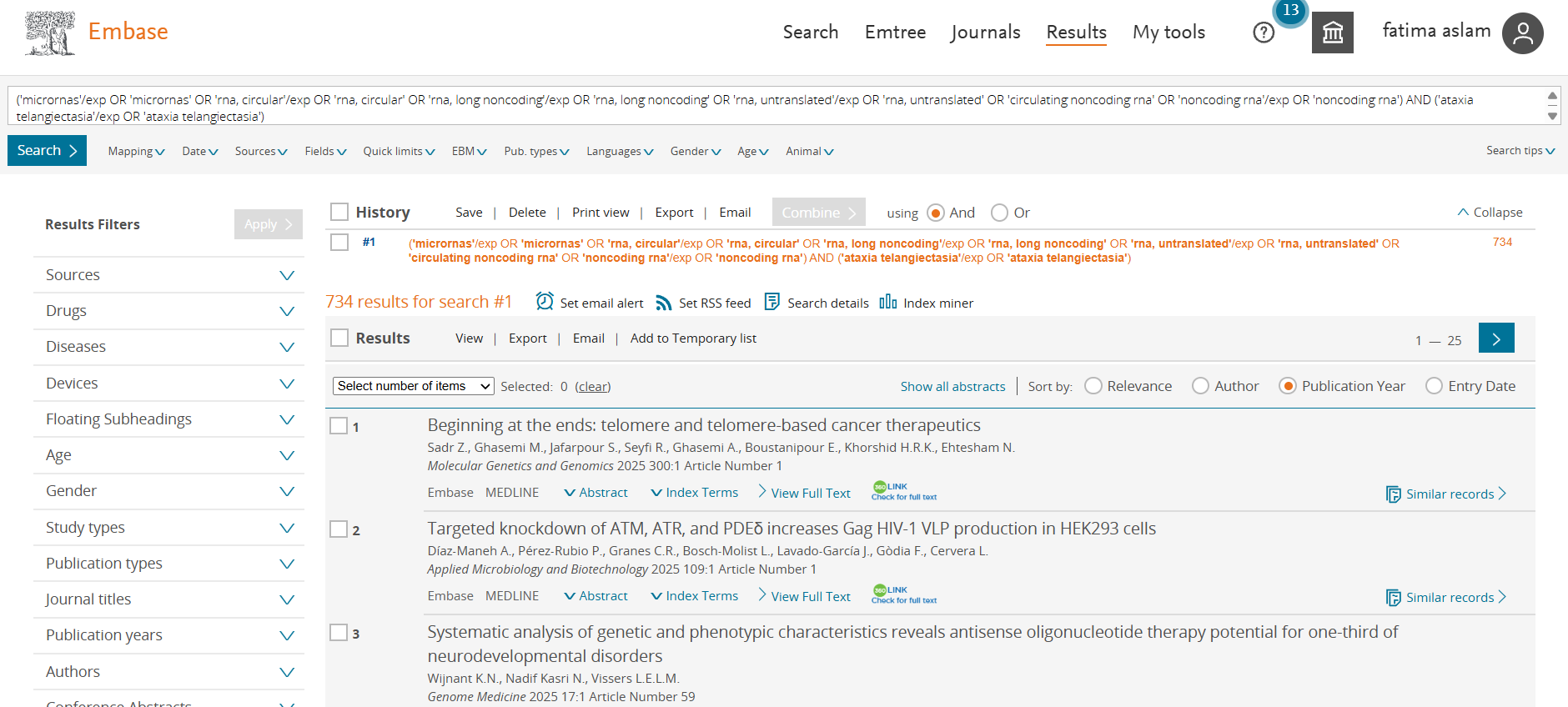
**
